# Integration of Web Analytics Into Graduate Medical Education: Usability Study

**DOI:** 10.2196/29748

**Published:** 2021-12-13

**Authors:** Jackson Massanelli, Kevin W Sexton, Chris T Lesher, Hanna K Jensen, Mary K Kimbrough, Anna Privratsky, John R Taylor, Avi Bhavaraju

**Affiliations:** 1 College of Medicine University of Arkansas for Medical Sciences Little Rock, AR United States; 2 Division of Trauma and Acute Care Surgery Department of Surgery University of Arkansas for Medical Sciences Little Rock, AR United States

**Keywords:** graduate medical education, website analysis, residency recruitment, medical education, website, analytics, usage, usability, user engagement, user-centered design, website design

## Abstract

**Background:**

Web analytics is the measurement, collection, analysis, and reporting of website and web application usage data. While common in the e-commerce arena, web analytics is underutilized in graduate medical education (GME).

**Objective:**

The University of Arkansas for Medical Sciences Department of Surgery website was revamped with input from in-house surgeons in August 2017. This study investigated the use of web analytics to gauge the impact of our department’s website redesign project.

**Methods:**

Google Analytics software was used to measure website performance before and after implementation of the new website. Eight-month matched periods were compared. Factors tracked included total users, new users, total sessions, sessions per user, pages per session, average session duration, total page views, and bounce rate (the percentage of visitors who visit a site and then leave [ie, bounce] without continuing to another page on the same site).

**Results:**

Analysis using a nonpaired Student *t* test demonstrated a statistically significant increase for total page views (before vs after: 33,065 vs 81,852; *P*<.001) and decrease for bounce rate (before vs after: 50.70% vs 0.23%; *P*<.001). Total users, new users, total sessions, sessions per user, and pages per session showed improvement. The average session duration was unchanged. Subgroup analysis showed that after the main page, the next 3 most frequently visited pages were related to GME programs in our department.

**Conclusions:**

Web analytics is a practical measure of a website’s efficacy. Our data suggest that a modern website significantly improves user engagement. An up-to-date website is essential for contemporary GME recruitment, will likely enhance engagement of residency applicants with GME programs, and warrants further investigation.

## Introduction

Web analytics is the measurement, collection, analysis, and reporting of website and web application usage data. Web analytics has been used in many industries [1-3] to understand and optimize their websites by improving user engagement and stimulating traffic [2-5]. These tools facilitate the measurement of a website’s efficacy using metrics, such as the bounce rate, which is the percentage of users who visit a site and then leave (ie, bounce) without continuing to another page on the same site [6]. The bounce rate represents a website’s effectiveness in encouraging users to continue their visit beyond the first page [7]. A high bounce rate implies that users are uninterested in the site’s content or that the design is unsuitable for the user [8]. The bounce rate and other web analytical tools are underutilized in graduate medical education (GME).

The bounce rate and other similar web metrics are commonly utilized in e-commerce and web-marketing to identify user trends, improve website flow and usability, and ultimately boost web traffic. Examples of the contemporary use of web analytics would be an e-commerce website measuring user traffic during a sale or a holiday to see which items garner the most attention, a software company using page metrics to direct visitors to help forums or FAQ sections faster, an educational website tracking user data to more easily provide information on admissions or courses to its students, and a political campaign measuring a candidate’s web traffic after a debate [9].

A high bounce rate is concerning for a site because it means visitors do not browse through the entire site and do not get a comprehensive look at all it has to offer [6], which generally indicates that the site or page is not relevant to its visitors. The bounce rate has been described as the measure of a user’s satisfaction with a given page [10], and most businesses agree that pages with high bounce rates should be reworked to be more user-friendly, with the goal of making a user’s visits more frequent and longer in duration, thus lowering the bounce rate [3]. Similarly, lowering the bounce rate is generally associated with improved user engagement by allowing the user to explore more content in a pleasing and persuasive fashion [3]. Reciprocally, studies have also demonstrated that higher user satisfaction lowered the bounce rate [11]. A high bounce rate could be due to a multitude of factors, but is primarily attributed to an illogical navigation scheme, irrelevant and disorganized content, or faulty design [3]. Our website redesign project aimed to address all of these issues.

It is well established that residency applicants find a GME program’s website a valuable tool in determining where to apply, where to interview, and how to rank individual programs [12]. In 2003, Mahler et al showed that 96.5% of emergency medicine applicants used the web as a resource to investigate residency programs, and almost all of the applicants applied to programs they thought had the best website [13]. Many individual residency and specialty programs have subsequently evaluated their own websites, creating a vast amount of literature on the subject [12-26]. These studies add to the overall subject of human-computer interaction (HCI), where web analysis has been studied extensively [27]. These studies found that while many applicants use a program’s website to gather information, the sites themselves lack all of the content items applicants are looking for (eg, faculty research and resident biographies), leaving room for improvement [12-26]. The purpose of our website is multifold. It gives users an introduction to our department, lists the organizational infrastructure, provides information about faculty and their research endeavors, acts as a recruitment tool by providing demographic info about the program and its residents, serves as a resource for undergraduate students, and, in some cases, provides clinical practice guidelines.

Our study investigated the use of web analytics to gauge the impact of our institution’s Department of Surgery website redesign project, which was done in close collaboration with in-house surgeons. While website redesign has been extensively studied in HCI research for the last 30 years [27], it does not seem to be as extensively studied or used in the GME setting. To our knowledge, no previous study has used analytical data to evaluate a GME website and very few [18,24] completed a follow-up assessment after redesigning their website to determine if changes to the website were effective. The purpose of this study was to utilize web analytics to gauge the impact of our department’s website redesign project and prove with objective metrics that a fresh and up-to-date website will improve web traffic for a GME website and thus will improve a GME program’s exposure to prospective applicants and will potentially help with residency recruitment. The objectives of this study were to (1) examine and analyze user data for our department website before and after the redesign and (2) assess if the implemented changes improved user engagement, web traffic, and throughput. The goal of our project was to determine if a fresh and modern GME website can be used as an effective recruiting tool.

## Methods

The Department of Surgery website for the University of Arkansas for Medical Sciences (UAMS) was redesigned during the summer of 2017 and launched on August 30, 2017. Using Google Analytics software, multiple variables were tracked to gauge the website’s performance. Google Analytics is a set of free online tools that track website visitors’ mouse clicks and information requests. The data gathered by Google Analytics can be used to see which pages on an organization’s website are the most popular or most accessed, what type of information visitors are interested in accessing, what path visitors take as they navigate to and away from an organization’s website, how much time they spend on the site, and a variety of other metrics [9]. These data are not stored on visitors’ computers and contain no personally identifiable information. Google Analytics works by having a website administrator attach code onto each server that hosts the web page, and begins tracking the page as soon as it is uploaded [9]. The Google Analytics code is easy to upload to a website’s server, and Google offers step-by-step instructions to facilitate implementation, making it an easily accessible option for any program that wants to track user or site metrics [9]. For our study, we examined monthly and aggregate user and webpage data to examine the effects of the redesign project. Data were compared for 8-month matched periods from January to August of 2017 and 2018, representing data from before and after the new site was launched, respectively. The months of September through December for each year were excluded to account for the seasonal effect of GME interviews adversely elevating website traffic.

The study population included all visitors to the website during the aforementioned time periods. No users were excluded. Users remained anonymous and were not individually tracked, and all collected data were completely deidentified.

The primary end-points examined were total page views and bounce rate. Secondary variables included total users, new users, total sessions, sessions per user, pages per session, and average session duration. The Google Analytics software package also provided a breakdown of the activity on each page of the website, allowing us to stratify which pages were the most heavily trafficked, and thus, which sections of the new website were the most popular among our users. An unpaired Student *t* test was used to test for significant differences among our variables. Continuous variables were evaluated using GraphPad Prism Version 8.0.0 (GraphPad Software, Inc). The project received a nonhuman subject research exemption from the UAMS Institutional Review Board (IRB #249934).

## Results

**Figure 1:**
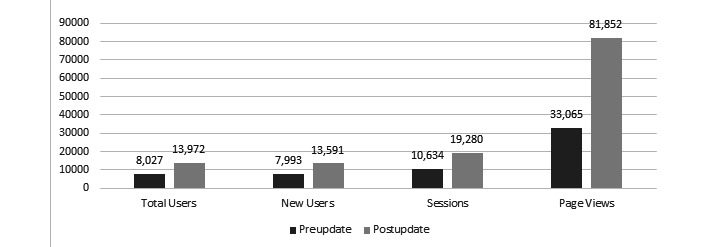
Graph demonstrating significant increases in total users, new users, total sessions, and page views after the website update.

**Figure 2:**
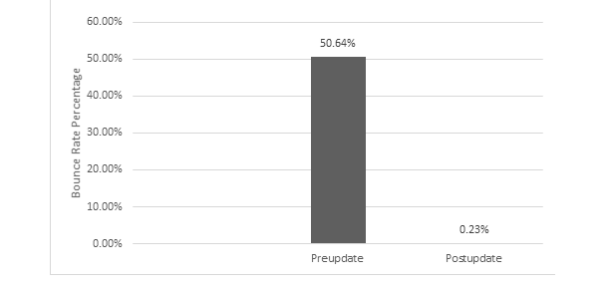
Graph demonstrating a significant decrease in the bounce rate after the website update.

**Figure 3:**
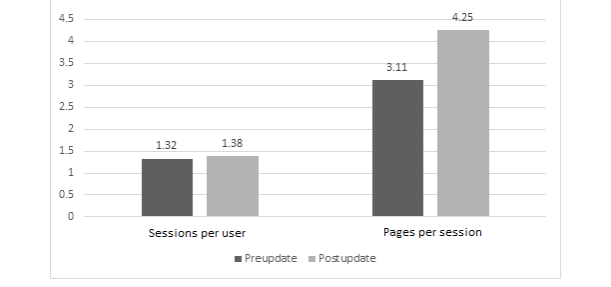
Graph demonstrating an increase in pages per session and similarity between sessions per user.

**Figure 4:**
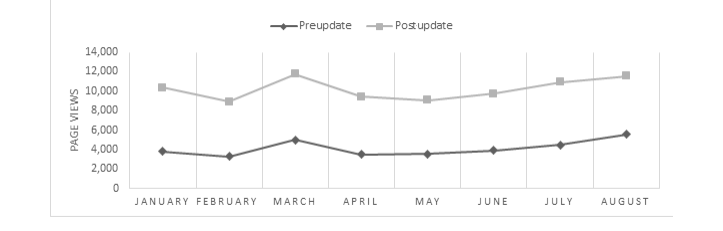
Graph comparing page views between 8-month matched periods before and after the website update.

After the departmental website redesign, improvements were observed in most of the tracked metrics, including both our primary end-points. A statistically significant increase in total page views (*P*<.001; Figure 1) and a statistically significant decrease in bounce rate (*P*<.001; Figure 2) were observed. There were also statistically significant improvements in the number of total users (*P*<.001; Figure 1), number of new users (*P*<.001; Figure 1), number of sessions (*P*<.001; Figure 1), average number of sessions per user (*P*=.004; Figure 3), and pages viewed per session (*P*<.001; Figure 3). The average session duration was unchanged. The chronological breakdown of total monthly page views for the 8-month matched periods preupdate and postupdate are shown in Figure 4.

Before the update, the top 5 most visited pages were (1) Home Page, (2) Our Residents, (3) General Surgery Residencies and Fellowships, (4) Residencies and Fellowships, and (5) Vascular Surgery. After the redesign, the top 5 most visited pages were (1) Our Residents, (2) Junior Clerkship Goals and Objectives, (3) Home Page, (4) Trauma Guidelines, and (5) Residencies and Fellowships.

## Discussion

### Principal Findings

After modernizing our department website, we noted significant improvements in virtually all tracked metrics. Utilizing Google Analytics, we were able to show a nearly 2.5-fold increase in the total number of page views and a statistically significant drop in our website’s bounce rate from 50.64% (N=10,634) to 0.23% (N=19,280) when comparing 8-month matched periods before and after the updated website was launched. Secondary analysis of the user data showed that the most commonly visited pages on the website were related to our undergraduate and graduate medical education programs.

Our previous department website received universally poor feedback for being outdated, incomplete, and difficult to navigate. Based on an institution-wide initiative to update the UAMS web presence, we performed a total overhaul of the Surgery Department website to improve the organization, flow, usability, and multimedia component of the site through the addition of pictures and videos. The principles used to guide the redesign were related to optimization of web content, frequent and consistent content updates, improving content accessibility and responsiveness, search engine optimization, and maintaining institutional branding. Overall, the new website’s content follows the Web Content Accessibility Guidelines (WCAG) 2.1 standard, which provides guidelines for the way content is presented on a website (eg, the way graphics and colors are used, column width, moving elements, text size, etc). Our website redesign plan allowed us to switch website templates to the new standard, which also presented an opportunity to update its content. Responsiveness is the ability of a site to adjust to different resolutions and devices in real time, for example, zooming in and out of the website on a mobile device. Older websites, including our previous website, are static and thus unable to accommodate for these differences, leading to a less favorable user experience. Our updated website, using the new WCAG 2.1 standards, is much better able to handle these differences, providing a more fluid user experience. Search engine optimization is the process of maximizing the number of visitors to a particular website by ensuring that the site appears high on the list of results returned by a search engine, such as Google and Yahoo. Search engine optimization is affected by the code of the template used to create the website, specific webpage content, and site organization. Branding refers to the overall theme used on a given site. Newer web templates are more stringent about the use of colors and logos in order to keep things consistent, reduce variation from page to page, and maintain the brand throughout a given website.

Some of the specific changes we made included the following: a detailed breakdown of each division within the department, including a comprehensive list of faculty within each division with titles and links to clinical bios; descriptions of each division’s current priorities, initiatives, and research endeavors with specific divisional guidelines in some cases; and a dedicated section for medical students, with detailed information on the M3 junior clerkship, M4 surgery electives, M4 surgery honors course, and Summer in Surgery program, an innovative program geared toward M1 students interested in pursuing a career in surgery. Regular prospective updates are made to the new site every few months to ensure the information is up-to-date, including the addition of new faculty members, faculty research endeavors, new clinical and operational guidelines, department news, and divisional updates.

While there is a multitude of research in HCI analyzing web traffic, there is a paucity of research in examining GME websites, especially in medical education literature. Much of the research evaluating GME websites up to this point has been qualitative, comparing a particular site to a predetermined checklist of features, either chosen by the investigators [14-25] or suggested by residency applicants [12,13,26]. Little work has been done to investigate how improvements to a program’s website impact the user experience or applicant’s perceptions of a program, and to the best of our knowledge, no studies have taken a quantitative data-driven approach to this process. Web analytic software provides GME programs the means to objectively evaluate their websites and the effect any improvements may have on end-users, such as residency applicants. Prior studies have demonstrated that Google Analytics is an effective tool to assess web traffic on health sites. Pang et al used Google Analytics to assess desktop and mobile website traffic for a health channel website. They examined traffic over a 3-month period and found that the most heavily visited pages were not the home page and search results like they hypothesized, concluding that website owners should examine their own web traffic to tailor their designs to their users’ diverse needs, search approaches, and behaviors [28]. They also examined users’ outgoing pages, which varied from the home/search page to other specific content pages, but they were not able to determine why they left the site on those pages (eg, the user found all the information, the user is going to look on another site, etc) [28].

Prior studies have evaluated GME websites in almost every specialty [12-25]. Embi et al published one of the first studies to show a program’s website is a critical tool for GME recruitment [12]. They surveyed a large cohort of residency applicants to a single internal medicine program and determined that internet-based tools were more viable options for information delivery than paper resources, such as printed brochures. They reported that 80% of applicants used websites to help decide where to apply, 69% used websites to determine where to interview, and 36% used websites to help rank order programs for The National Resident Matching Program [12]. Mahler et al surveyed emergency medicine residency applicants and showed that online information from programs’ websites influences an applicant’s decision by highlighting aspects of the program that are pertinent to the applicant and making them easy to find; 40% of applicants rated an easily navigated website as “very” or “moderately important” to their decision-making process [13]. They hypothesized that an easily navigated complete website may improve recruitment to emergency medicine residency programs.

Our study found that after the redesign, there was a significant increase in web traffic to the website. Google Analytics software showed improvements in virtually all tracked metrics, including total page views, total users, new users, sessions, sessions per user, pages viewed per session, and most importantly bounce rate. We suspect that the improved metrics of the new site are due to better flow and organization, ease of navigation, and being much more user friendly. The significance of this tremendous drop in the bounce rate cannot be understated. Prior to the redesign, approximately 50% of users visited the first page and immediately left (“bounced”), not interacting with any other portion of the site, and after the update, only 0.23% of all users bounced, meaning 99.77% of users visited a subsequent page on the site. While we cannot directly attribute the improved website metrics to any particular change and, at present, cannot directly tie the improved web traffic to improved residency recruitment, based on feedback about the old site from students, residents, and faculty, we postulate that the new website’s ease of use and fresh look played a very direct role in improving user experience and user engagement, as evidenced by the tremendous drop in the bounce rate. We hypothesize that this improved user engagement and web traffic will ultimately translate downstream to improved GME recruitment, which is the focus of the next phase of this project.

Based on the above data, we believe the implications for GME are clear. As e-commerce sites must attract new users (ie, shoppers) to make sales, GME programs must make themselves attractive to residency applicants (ie, “the consumer”). The program or department website is often the first contact a visitor has with a program and its first opportunity to sell itself to the prospective applicant; thus, having a well-designed and visually appealing website is essential to making an impactful first impression, particularly for someone who is not familiar with the program. An easy-to-use website with an abundance of information applicants are looking for gives a program credibility and makes it more appealing to the “buyer” (ie, the applicant). We predict this positive first impression could help persuade applicants to think more favorably about a program, particularly those they may not have otherwise considered. We hypothesize that programs with a modern website will improve their web metrics, which will translate to increased engagement of users with these programs and enhanced opinions of the programs by applicants. For programs that focus attention on their web presence and utilize web analytics in this way, we theorize this will ultimately lead to improved match rates and matching of preferred applicants. Based on our experience, we believe that using web analytics to track user data is an important but underutilized tool that can help GME programs identify areas of weakness and optimize their web presence to make themselves more marketable to prospective GME applicants. Without a focused and deliberate effort in this regard, contemporary GME recruitment is inadequate. We believe that a program with a modern website that focuses attention on web presence and utilizes web analytics will improve metrics, which will translate to increased user engagement, enhanced opinions of these programs by applicants, and ultimately improved match rates and matching of preferred applicants. With respect to our own GME program, the numbers of applicants to our program before the update were 773 (2016) and 996 (2017). After the update, we had 793 (2018), 871 (2019), and 1059 (2020) applicants (unpublished data). While there was no immediate boost in the number of applicants after the update, the number of applicants has steadily increased since then. This increase could be due to a number of factors, many of which are outside the scope of this study, but based on the improved web metrics of the new website and the results from a follow-up study we conducted surveying applicants to our program about their opinions and experiences with the new website, we firmly believe the new website played a positive role in driving applicant numbers up. In that study (N=121), the results of which have not yet been published, the majority (98.3%) of interviewees visited our department website before their interview day, the highest-rated features of our site were easy navigability and clean design (57.9%), the majority (64.5%) of applicants reported that the department’s website influenced their opinions of a program, and the majority (94.2%) of applicants reported that a well-developed user-friendly department website is an important factor in selecting a residency program (unpublished data).

In a previous study that assessed the quality of general surgery residency program websites for accessibility, ease of use, design, and content, among the 167 program websites evaluated, an average of 6 out of 16 content items were present and 6 out of 10 design principles were followed, when compared to established principles of website design and content [21]. To address this concern with our site, as part of the secondary analysis, we examined our usage data to track which subpages had the heaviest traffic and lowest bounce rates. Prior to the redesign, the most popular page on the site was the “Home Page,” followed by “Our Residents,” “General Surgery Residency and Fellowships,” “Residencies and Fellowships,” and “Vascular Surgery.” After the update, the most popular pages were “Our Residents,” “Junior Clerkship Goals and Objectives,” “Home Page,” “Trauma Guidelines,” and “Residencies and Fellowships.” Based on this analysis, it is readily apparent that the majority of visitors to the site are looking for information about our undergraduate and graduate medical education programs, which allowed us to tailor the site to the needs and desires of our users. The improved usability and updated information have helped increase traffic 2.5 fold, which we hypothesize will improve our ability to attract and recruit residency applicants. Previous studies have shown that what applicants prioritize on a program’s website varies between specialties, so we suggest that GME programs should survey their applicant pool to determine where to focus their attention and use web analytic tools to follow user traffic and bounce rates. By doing so, they can optimize their web presence and leverage their department and program websites as powerful recruiting tools to attract prospective applicants. For all these reasons, we feel strongly that an organized and up-to-date department or program website is an essential component of contemporary GME recruitment.

### Limitations

One limitation of our study is that we did not directly survey visitors to gauge their opinions about our website in real time. While there were significant improvements in virtually all of our tracked data points after the update, which we attribute to the improved website design, this study did not determine which specific changes were responsible for the improved traffic. We also cannot say with certainty that the changes to the website were responsible for the improved user traffic, but we have no other plausible explanation for this other than the updated website.

### Conclusion

Web analytics is a quick and practical measure of a website’s efficacy. Our data suggest that a fresh and modern website significantly improves user engagement, web traffic, and throughput. Based on these data, we hypothesize that a polished and up-to-date website is essential for contemporary GME recruitment, will enhance engagement of residency applicants with GME programs, and warrants further investigation.

## Abbreviations

GME: graduate medical education
HCI: human-computer interaction
UAMS: University of Arkansas for Medical Sciences
WCAG: Web Content Accessibility Guidelines
